# Sport-specific variability in the energy cost of constant speed running: Implications for metabolic power estimations

**DOI:** 10.1371/journal.pone.0329323

**Published:** 2025-08-06

**Authors:** Jan Venzke, Robin Schäfer, Petra Platen

**Affiliations:** Department of Sports Medicine and Sports Nutrition, Ruhr University Bochum, Germany; Ordu University, TÜRKIYE

## Abstract

**Introduction:**

Metabolic power is essential for assessing the physical demands of team sports. Accurately determining the energy cost of constant speed running (EC_0_), is crucial for refining these. EC_0_ depends on factors like running velocity and V̇O₂max and varies between athlete groups due to training adaptations and sport-specific body characteristics. To ensure accurate energy expenditure, EC_0_ should be individually determined based on the specific team sport, improving player monitoring, recovery, and load management.

**Materials and Methods:**

An experimental cohort study collected data from 339 incremental treadmill tests in elite team sports athletes: 11 male handball players, 120 male soccer players, 23 male and 185 female field hockey players. Athletes performed a treadmill protocol to exhaustion while O_2_-uptake, CO_2_-output, respiratory exchange ratio and ventilation were measured breath-by-breath. Data processing verified steady-state conditions. Net EC_0_ was calculated as energy expenditure above rest divided by velocity. Sport, speed, sex and V̇O_2max_ were defined as fixed effect variables.

**Results:**

Our random intercept and slope model with all predictors performed best. Handball players had the highest EC_0_ (estimated total mean) with 4.04 J/kg/m (CI_95%_ 3.88, 4.20), field hockey players with 3.95 J/kg/m (CI_95%_ 3.90, 4.00) and soccer players with 3.79 J/kg/m (CI_95%_ 3.73, 3.85). For the whole group, EC_0_ showed a curvilinear dependence on speed: increasing with speed up to ~3.5 m/s and then remaining relatively constant at higher velocities. However, grouping athletes by similar treadmill performance, EC_0_ remained constant across speeds.

**Discussion:**

Our data show multiple predictors must be considered to determine an appropriate EC₀ for each athlete. Although EC₀ remains stable across velocities for individuals, it varies significantly between sports and V̇O₂max levels, highlighting the need for individualized assessment. Calculating EC₀ per athlete may improve energy cost estimations, enhance the metabolic power approach and allow for more accurate analysis of metabolic data based on positional tracking.

## Introduction

Monitoring and managing physical load in team sports has become a critical aspect of optimizing performance and minimizing the risk of injury [1]. In team sports, which are often characterized by intense physical demands such as high-speed sprints, jumps and frequent collisions, understanding the physical demands is crucial for both coaching strategies and player well-being. Modern metrics such as ‘metabolic power’, derived from player tracking data, have emerged as valuable tools for accurately assessing the physical demands placed on players [2–4]. These parameters offer insights beyond traditional measures of speed and distance, providing a multi-dimensional understanding of the metabolic load experienced by players during competition and training [5].

Technological advancements in wearable tracking devices have transformed sports analytics. In team sports, where explosive action is essential, wearables provide insights into acceleration, deceleration and directional changes – data that cannot be obtained through observation alone. This technology allows detailed analysis of external loads, making metrics such as metabolic power a reliable indicator of player adaptation to match and tournament demands. Metabolic power is calculated by estimating the instantaneous energy cost of accelerated running based on speed and acceleration data. The energy cost of running at constant speed (EC_0_) serves as a baseline value, which is adjusted on changes in speed (equivalent slope) and body mass dynamics. This allows continuous estimation of energetic demands throughout a match or training session using positional data alone. While EC_0_ is a metabolic baseline parameter derived from steady-state oxygen uptake at constant running speeds, it should not be confused with mechanical energy cost or total energy expenditure. Metabolic power, as used in positional data analysis, builds upon EC_0_ by incorporating instantaneous speed and acceleration.

Metabolic power is a useful metric for assessing the intensity of match play in team sports, as it considers both speed and acceleration. Acceleration is more energetically demanding than maintaining speed [6] and the metabolic cost varies with initial speed [3]. Accelerations and decelerations are also physiologically relevant in team sports even at submaximal speed [7], and are considered to be among the most demanding elements in team sports, contributing directly to overall energy expenditure [2,8]. Therefore, the combination of speed and acceleration provides a more comprehensive view of energy expenditure than speed alone in team sports [3,5,9–11]. For example, two players covering the same distance may have vastly different metabolic loads depending on the number and intensity of accelerations, which traditional distance-based metrics would fail to capture.

When the metabolic power approach was first introduced [2], the energy cost of constant speed running was set at 3.6 J/kg/m based on the results of an earlier study of endurance mountain runners [12]. Since then, the energy cost of constant speed running has been renamed EC_0_ [13] and researchers are advised to determine EC_0_ for each individual subject [13] or to use a unique value based on the subject group. The energy cost of running is traditionally determined by steady-state oxygen consumption at constant speed divided by the running speed. This method has been validated by studies showing consistent results across different approaches [14,15]. The energy cost per unit distance remains relatively constant across different running speeds, suggesting that the metabolic cost does not change significantly with speed. However, the mechanical energy cost tends to decrease with increasing speed [16]. The energy cost of running may vary between different types of athletes. For example, marathon runners tend to have a lower energy cost for steady running compared to soccer players, probably due to their specific training adaptations such as a more economical running style [17]. However, there is a lack of knowledge about the EC_0_ values of different groups of athletes. Since EC_0_ is directly used in the calculation of metabolic power, inaccuracies in this value can systematically affect the estimation of energy expenditure. This makes it essential to determine sport- or individual-specific EC_0_ values for accurate load monitoring.

Running economy, defined as the energy required for a given submaximal running velocity, is a key determinant of distance running performance. It is influenced by physiological factors such as metabolic efficiency (increased mitochondrial density and oxidative enzymes), cardiopulmonary efficiency (reduced oxygen transport workload) [18,19] and muscle-tendon properties (improved elastic energy storage through increased muscle stiffness) [20,21]. Biomechanical factors also play a role, including optimal stride length, reduced vertical oscillation, efficient leg mechanics, midfoot strike, and coordinated muscle activation with minimal coactivation of antagonistic muscles. Environmental factors can also influence running economy [21].

The aim of this study was to investigate the cost of constant speed running in different types of sports (mainly team sports), at different speeds on a treadmill. We hypothesized that [1] there is a difference of EC_0_ across different types of sports and that [2] EC_0_ is dependent on different predictors such as running velocity and individual V̇O_2_max. By analyzing this metric, we aim to provide a more accurate assessment of the energy requirements of team sports as calculated and given by positional data alone. Specifically, monitoring player performance, training recovery and load management in team sports will benefit from a more accurate assessment of energy expenditure during a team sport match or training session.

## Materials and methods

### Study design

An experimental cohort study was carried out. Data were collected during incremental treadmill tests in the laboratories of the Department of Sports Medicine and Sports Nutrition of Ruhr University Bochum from 2015–2024 and were accessed on the 10^th^ of December 2024. The Ethics Committee of the Faculty of Sport Science at Ruhr University Bochum reviewed the study protocol (File number: EKS S 2025_04) and confirmed that no formal ethical approval was required for this type of secondary data analysis. All participants had provided written consent for the use of their performance diagnostic data for scientific purposes. All data were fully anonymized prior to analysis, and the authors had no access to information that could identify individual participants.

### Participants

In total, data from 339 tests were collected, originating from 220 individual athletes. This included 11 elite male handball players (11 tests), 23 elite male field hockey players (23 tests), 100 elite female field hockey players (185 tests), and 86 elite male soccer players (120 tests). 81 athletes completed multiple measurements (2–6 tests) at different time points. Anthropometric data (height, weight) and age were collected before the running protocol. Handball players were tested in 2024, male field hockey players were tested in 2016, female hockey players were tested between 2016 and 2024, and soccer players were tested between 2015 and 2019. The handball player competed in the second-highest national league in Germany, the soccer players were active in the first and second divisions of the German Bundesliga, and the field hockey athletes competed at the highest competition level in Germany. All athletes trained and competed at a high-performance level and were categorized as elite based on their training volume and competitive status.

### Instruments

The athletes performed one of three incremental treadmill tests (h/p/cosmos Saturn 250/100) to exhaustion. The specific protocol used was predetermined by the participating clubs or institutions as part of their internal fitness assessments. Therefore, athletes were not randomly assigned to protocols, and the choice of protocol often coincided with the type of sport. Each athlete completed only one protocol, with only two individuals participating in more than one. As such, protocol and sport were largely confounded, making it statistically infeasible to isolate the effect of protocol in the analysis. Protocol A started at 2.5 m/s, each step lasted 5 min and the increment was 0.5 m/s. Protocol B started at 2.0 m/s, each step lasted 5 min and the increment was 0.4 m/s. Finally, protocol C started at 2.22 m/s (8 km/h), each step lasted 3 min and the increment was 0.55 m/s (2 km/h). The treadmill was set at a 1% incline to better simulate the energy cost of running outdoors by compensating for the lack of air resistance [22]. For athletes with repeated measurement, the same protocol was used in all tests to ensure consistency withing subjects.

Oxygen uptake (V̇O_2_, ml/min), CO_2_ output (V̇CO_2_, ml/min), respiratory exchange ratio (RER) and ventilation were measured breath-by-breath (MetaMax 3B-R2, Cortex Biophysik GmbH, Leipzig, Germany). The spirometer has previously been reported to have adequate validity and reliability for field testing [23,24]. The spirometer was calibrated using a 3-litre gas flow pump and a standard gas (oxygen, 15.00%; carbon dioxide, 5.09%) according to the manufacturer’s recommendations.

### Data processing

Raw breath-by-breath data were processed using a moving average of 1 s. The energy cost per time frame was calculated using the approach of McArdle et al [25] by multiplying the oxygen uptake by the corresponding caloric equivalent determined by the respiratory exchange ratio. Steady-state conditions for each step were verified by ensuring that the percentage deviation in V̇O_2_ over 30 seconds was less than 5%. A stage was only included if it was completed. Trials were also excluded if the subject was unable to run to maximal exertion due to injury or similar. Energy cost was then calculated in J/kg/m for each step to determine EC_0_ in steady-state conditions for different running speeds. Net cost was calculated as the rate of energy expenditure above rest (assumed to be 3.5 ml/kg/min) [25] divided by speed [26].

### Statistical analyses

All statistical analyses were performed with JASP (0.19.3).

Different linear mixed models were applied and compared to analyse the relationship between EC_0_ and its different hypothesised predictors (V̇O_2max_, velocity, type of sport). EC0 was the dependent variable, while V̇O2max, speed, and type of sport were defined as fixed effect variables. Repeated measurements per athlete were accounted for by including “Subject ID” as a random-effects grouping factor. Sex differences were only analyzed for field hockey players due to absence of female athletes in soccer and handball. To model the relationship between EC₀ and its predictors, we used a two-level linear mixed-effects model. At Level 1 (within subjects), EC₀ for each test occasion *i* and subject j was predicted by sport, running velocity, and individual V̇O₂max:


$$𝐄{𝐂}_{oij}=\beta_{oj}+\beta_{1j}\;Spor{𝐭}_{1j}+\beta_{2j}Velocit{𝐲}_{1j}+\beta_{3j}\dot{𝐕}0_{2}ma{𝐱}_{ij}+\varepsilon_{ij}$$

At Level 2 (between subjects), intercepts and slopes were allowed to vary across individuals to account for inter-individual differences in the strength of these associations:


$$\beta_{0𝐣}=\gamma_{00}+{𝐮}_{0j}$$

$$\beta_{1𝐣}=\gamma_{10}+{𝐮}_{1𝐣}$$

$$\beta_{2𝐣}=\gamma_{20}+u_{2𝐣}$$

$$\beta_{3𝐣}=\gamma_{30}+{𝐮}_{3𝐣}$$

Here, the γ terms represent fixed effects across the population, while the u terms reflect subject-specific deviations. The residual error term εᵢⱼ was assumed to be normally distributed with constant variance.

To compare model fit, we used several statistical criteria. The Akaike information criterion (AIC) and the Bayesian information criterion (BIC) were employed to balance model fit with parsimony, while deviance and log-likelihood provided complementary measures of absolute fit. AIC was the primary criterion guiding model selection due to its balance between goodness of fit and generalizability. Among the tested models were simpler specifications (e.g., velocity only), pairwise combinations of predictors (e.g., sport + velocity), and more complex models including body mass. The final model including sport, velocity, and V̇O2max yielded the best performance across all fit criteria and was there therefore retained. Based on theoretical considerations and visual inspection of the data, we tested for non-linear effects of running velocity by including a quadratic term (velocity²) as an additional fixed effect. We also compared the estimated means with the 95% confidence intervals of our models. To assess the influence of using a fixed resting metabolic rate, a sensitivity analysis using gross EC_0_ was conducted (i.e., without subtracting 3.5 ml/min/kg). This model used the same fixed and random structure as the main model. Model comparisons are reported in the supplement (S1 Table).

## Results

Data sets from 339 tests were included. 13 trials were excluded due to premature termination of the trial due to injury. The anthropometric data of the sample are shown in Table 1. Handball players were the tallest and heaviest, but had the lowest mean relative V̇O_2_max of the male population. Male field hockey and soccer players had the highest relative V̇O_2_max (Table 1).

**Table 1:** Anthropometric data of the sample (mean ± SD).

	Field hockey players				Soccer players		Handball players	
	m		f		m		m	
n	23		185		120		11	
Height (m)	184.2	7.2	169.5	5.6	182.4	6.3	187.3	6.6
Weight (kg)	79.6	6.9	64.1	6.5	77.2	6.8	81.8	9.9
Age (y)	26.0	3.6	22.7	3.6	25.3	4.5	26.3	4.3
V̇O_2_max (ml/min/kg)	56.4	3.7	48.5	4.5	56.7	4.5	49.8	3.0

All field hockey and handball players (male and female) completed protocol A. A total of 29 soccer players participated in protocol B, while 91 players underwent protocol C. A total of 81 players from the total sample completed two tests; among them, 22 completed three tests, 11 completed four tests, 4 players completed five tests, and one individual completed 6 tests. We decided not to use the protocol as a predictor because only 2 athletes completed 2 different protocols.

**Fig 1:**
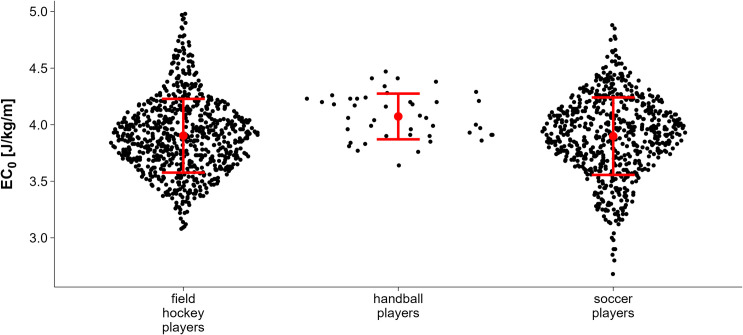
Energy cost of constant speed running in athletes from different team sports (total mean ± 95% confidence interval). Values represent observed group means based on raw data, not model estimates.

**Fig 2:**
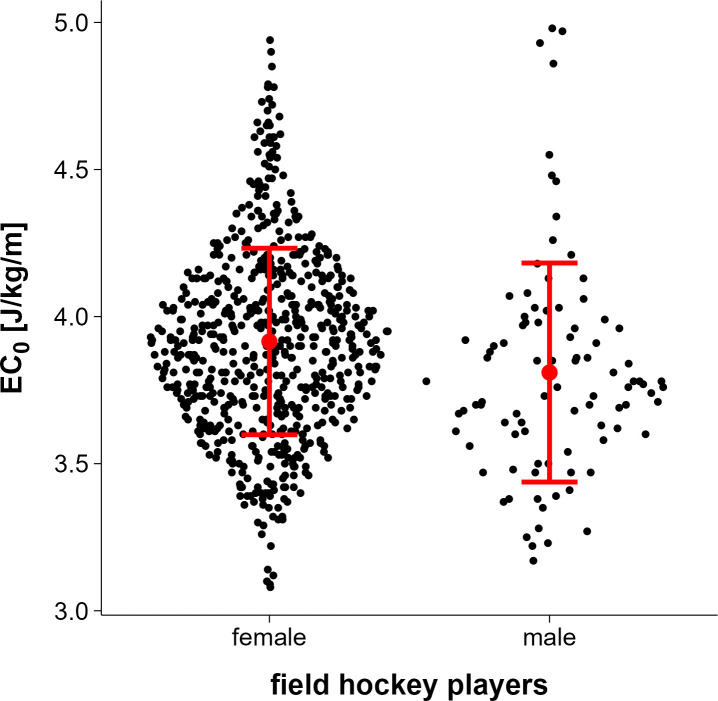
EC_0_ for female and male field hockey players (total mean ± 95% confidence interval).

Handball players had the highest total mean EC_0_ (4.07 J/kg/m CI_95%_ 3.94,4.20), followed by field hockey players (3.90 J/kg/m CI_95%_ 3.86, 3.95), and soccer players (3.90 J/kg/m CI_95%_ 3.84, 3.96) (Fig 1). These values represent observed group means derived from the raw data and are not based on model estimates. All EC₀ values reported refer to net energy cost, calculated by subtracting resting metabolic rate (assumed to be 3.5 ml/kg/min) from gross oxygen uptake at each stage. Female field hockey players had a higher total mean EC_0_ (3.92 J/kg/m CI_95%_ 3.87, 3.97) compared to males (3.81 J/kg/m CI_95%_ 3.65, 3.97) (Fig 2).

**Fig 3:**
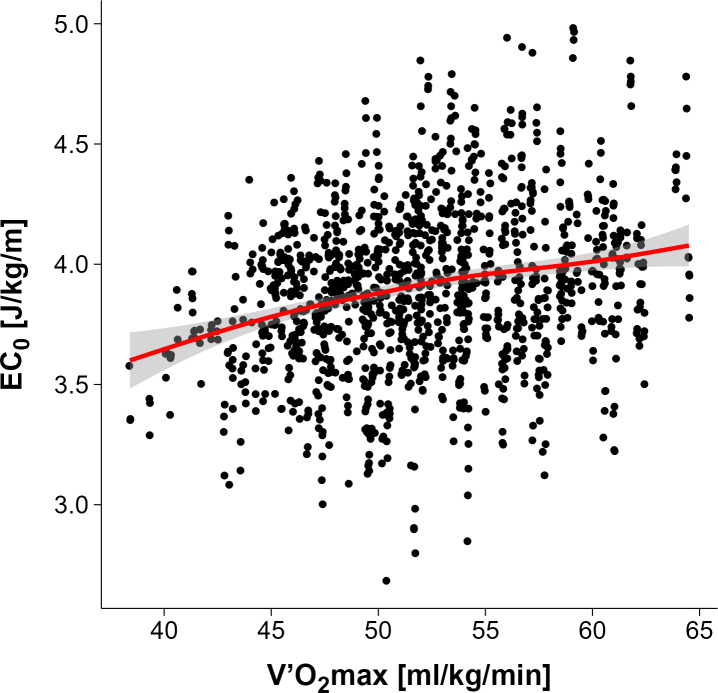
Relationship between maximum oxygen consumption and energy cost of constant speed running.

**Fig 4:**
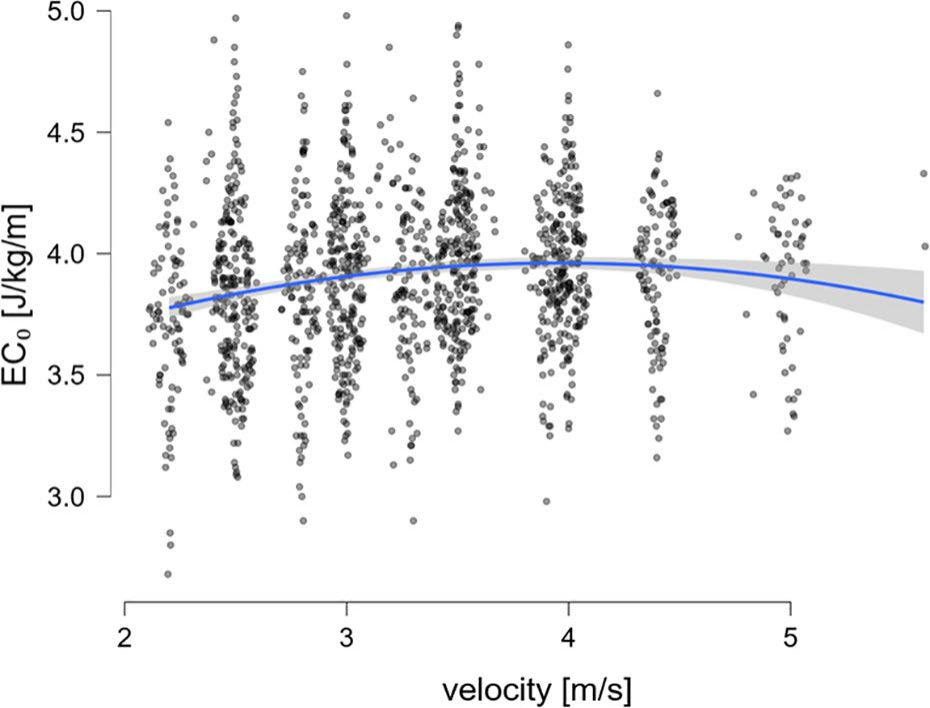
Energy cost of constant speed running as a function of velocity.

**Fig 5:**
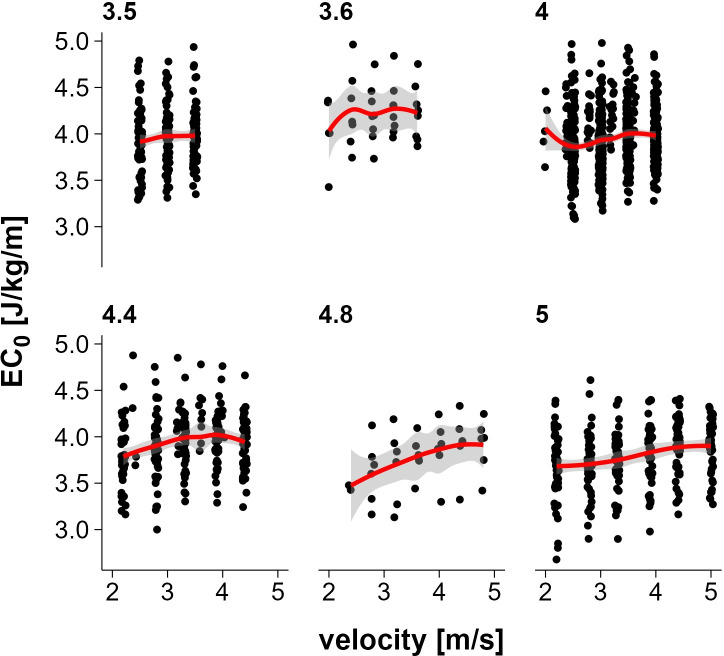
Energy cost of constant speed running versus running velocity grouped by maximum speed achieved. Subjects are categorized according to the highest velocity successfully achieved during the running protocol.

For the whole group of n = 339 tests, the linear model predicted a 0.3 J/kg/m higher EC_0_ per 10 ml/min/kg increase in V̇O_2_max (CI_95%_ 0.26–0.34) (Fig 3). EC_0_ increased with velocity, but at a diminishing rate. The positive linear effect (b = 0.542, p < 0.001) and significant negative quadratic effect (b = −0.071, p < 0.001) indicate a curvilinear relationship, with the steepest increases at lower speeds and saturation at higher velocities (see Fig 4). Analysis of the subgroups of athletes who each ran the same maximal speed to the end showed that in the 3 groups with a lower maximal speed, EC_0_ values were almost constant across different speeds. However, in the 3 groups with a higher maximal velocity, EC_0_ increased slightly with increasing running velocity (see Fig 5).

**Fig 6:**
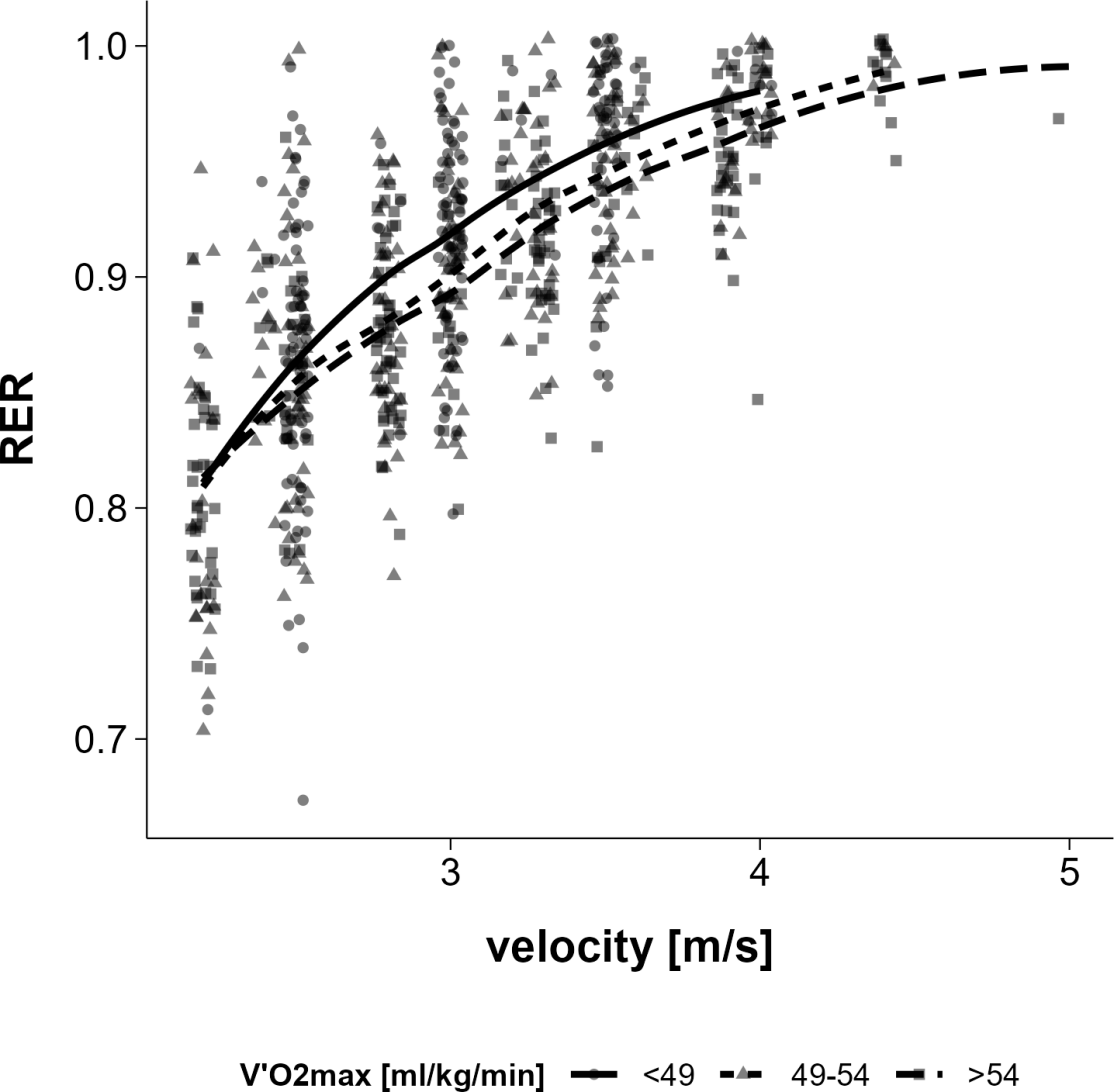
Respiratory exchange ratio (RER) as a function of velocity across 3 V̇O_2_max categories (low (<49 ml/min/kg), medium (49-54 ml/min/kg), and high (>54 ml/min/kg)).

When the sample was divided into three subgroups based on their V̇O_2_max (low (<49 ml/min/kg), medium (49–54 ml/min/kg), and high (>54 ml/min/kg), it was observed that athletes with a higher V̇O_2_max had a lower respiratory exchange ratio at each speed level (Fig 6).

To analyze the relationship between EC0 and its predictors, we used a linear mixed-effects model with sport, V̇O_2_max, velocity, and a quadratic velocity term (velocity²) as fixed effects, and participants ID as a random intercept with a random slope for velocity. This model performed best across all fit criteria (deviance: −266.9; log. Likelihood: 133.5; AIC: −246.9; BIC: −195.3) and was therefore retained for further interpretation. A sensitivity analysis using gross EC0 (i.e., without subtracting resting V̇O_2_) yielded nearly identical results and model fit (deviance: −268.0; log. Likelihood: 134.0; AIC: −248.0; BIC: −196.4), supporting the robustness of the findings. Full results for each net and gross EC_0_ models are available in the Supplement (S1 Table). Handball players had the highest EC_0_ (estimated total mean) with 4.04 J/kg/m (CI_95%_ 3.87, 4.20), field hockey players with 3.95 J/kg/m (CI_95%_ 3.90, 4.00) and soccer players with 3.79 J/kg/m (CI_95%_ 3.73, 3.85). Pairwise comparisons showed that EC_0_ was significantly higher in handball players compared to soccer players (p < 0.001), and also higher in field hockey players compared to soccer players (p = 0.015). No statistically significant difference was found between handball and field hockey players in the full sample (p = 0.664). When the analysis was restricted to male athletes, EC_0_ was again significantly higher in handball compared to soccer players (p < 0.001) and also compared to field hockey players (p < 0.01). The difference between soccer and field hockey players was not significant in this subgroup (p = 0.359), suggesting that the difference observed in the full sample may partly reflect the inclusion of female athletes in field hockey only.

Sex differences were found in field hockey players. In this subgroup, our model including V̇O_2max_, velocity, velocity² and sex as predictors performed best (deviance: 192.6; log. Likelihood: 96.3; AIC: −174.6; BIC: −133.3) among models with fewer predictors. The estimated marginal mean in EC_0_ for male field hockey players was 3.63 J/kg/m (CI_95%_ 3.52, 3.74) and for female players 3.94 J/kg/m (CI_95%_ 3.89, 3.99).

## Discussion

The main results of the study are the demonstration of the dependence of EC_0_ on the type of athlete from different sports, and the demonstration of a further dependence of EC_0_ on running velocity and the individual V̇O_2_max of the athlete. The differences in EC_0_ lead to the verification of hypothesis [1] and the knowledge of a dependence of different predictors verifies the secondary hypothesis.

### Energy cost of constant speed running

The estimated total mean values of the term for constant speed running (EC_0_) differed between the types of sport studied and their respective athletes. It was considerably lower than in other studies with similar types of athletes. Although there are no studies evaluating a constant speed running term for field hockey and handball players, they could be considered as ‘physically active’ comparable to Buglione et al. (2013) [26].

Soccer players in our study had an energy cost of 3.79 J/kg/m for constant speed running, which is lower compared to the literature (4.53 J/kg/m, [27], 4.64 J/kg/m, [3], 4.42 J/kg/m, [26], 4.19 J/kg/m, [17], 4.66 J/kg/m, [28], 4.19 J/kg/m, [29]) and more similar to the originally proposed value for EC_0_ for experienced mountain runners (3.6 J/kg/m, [12]). Several methodological differences may explain this discrepancy. Firstly, whereas previous studies mainly included amateur or sub-elite soccer players [27,29] or no soccer players at all [3], our study focused exclusively on elite players, who may exhibit superior running economy due to advanced physiological adaptation and technical efficiency [30,31]. Second, unlike studies conducted on artificial turf [27] or natural grass [28,29], we conducted our assessments on a calibrated treadmill, which minimizes surface-related energy losses and provides more consistent running conditions. The absence of external factors such as surface friction and ground compliance may have contributed to the lower energy cost observed in our study. Running on natural grass or artificial turf introduces variability in friction, which can increase muscular effort during propulsion and braking [32]. In addition, differences in ground compliance (i.e. surface stiffness and energy absorption) affect running mechanics, with softer surfaces requiring greater muscular compensation to maintain speed [33]. In contrast, treadmill running provides a consistent, relatively stiff surface with minimal frictional resistance, potentially reducing energy expenditure [21,34]. Thirdly, differences in footwear and running kinematics may have played a role. In contrast to field studies [27–29] where players wore soccer cleats or special cleats for artificial turf, treadmill running is typically performed in running shoes, which may improve running economy due to better cushioning and energy return [35,36]. In addition, research has shown that treadmill running may alter gait mechanics, for example by helping to bring the supporting leg back under the body during the support phase, which may affect the metabolic cost [37]. Finally, environmental factors also need to be considered. In contrast to outdoor studies where temperature, humidity, and wind resistance may influence energy expenditure [38], our study was conducted in a controlled indoor environment, minimizing external physical stressors. Given that heat and humidity can increase cardiovascular stress and energy expenditure [39], conducting our study in a standardized laboratory may have further contributed to the lower metabolic cost observed.

The higher running economy of soccer and field hockey players compared to handball players is likely due to their sport-specific endurance training adaptations. Soccer and field hockey players commonly engage in high-intensity interval training (HIIT) and small-sided games (SSG), both of which improve maximal aerobic speed (MAS), running economy, and velocity at lactate threshold, while having little effect on neuromuscular performance [40]. These methods optimize their energy efficiency during sustained running, contributing to their lower energy cost per metre compared to handball players. In contrast, handball players perform more frequent accelerations, decelerations and multidirectional movements which are likely to result in a less economical style when tested at constant speeds. In addition, handball players engage in more strength and power training to improve handball-specific performance, resulting in greater muscle mass and higher energy expenditure during forward running [41].

V̇O_2max_ is widely recognized as a key indicator of aerobic capacity, but it does not necessarily determine running economy. V̇O_2max_ reflects the maximum amount of oxygen an individual can use during intense exercise. Running economy, on the other hand, represents the oxygen cost of maintaining a given running speed. Our data show a positive association between EC_0_ and V̇O_2max_ which is consistent with previous research suggesting that higher aerobic capacity may be accompanied by reduced metabolic running economy [42–44]. Athletes with a higher V̇O_2max_ have a lower mean running economy (higher EC_0_) during an incremental treadmill test to exhaustion (Fig 3). This is at least in part due to their higher fatty acid oxidation (lower RER) at all running speeds (Fig 6), which in turn reduces metabolic running economy [45]. A reduction in (metabolic) running economy has also been shown after a period of training that resulted in an increase in V̇O_2max_ [42]_._ On the other hand, previous studies reported that the subgroup of athletes with the highest maximal speed during the incremental treadmill tests, which are probably those with the highest values of V̇O_2max_, had the lowest EC_0_ values (highest running economy) compared to the athletes with lower maximal running speed. This may be due to better biomechanical adaptations in the high V̇O_2max_ group [18].

In the subgroup of field hockey players, we were able to show that sex has an effect on EC_0_, with the female field hockey players having a slightly higher EC_0_ value compared to the male group of players. This is in line with previous research, showing that male athletes generally have better running economy than female athletes at absolute running speeds, i.e., they consume less oxygen per unit of body weight at the same speed [46,47]. However, when running intensity is matched relative to individual capacity, and oxygen consumption is expressed in ml/km/kg in experienced runners, these differences disappear [47,48]. The latter one is in contrast to our findings, as the small difference in the mean EC_0_ of 0.11 J/kg/m between female and male hockey players actually increased to an estimated mean of 0.31 J/kg/m when individual V̇O_2max_ was included in the model. This suggests that the lower running economy in female field hockey players may be due to biomechanical rather than metabolic factors. This difference between the group-level mean EC₀ (3.95 J/kg/m) and the sex-specific model estimates (3.94 for female and 3.63 for male players) can be explained by the model structure. In the group-level model, sex was not included as a predictor, resulting in an averaged estimate across all hockey players. In contrast, the sex-specific model accounted for individual differences in both V̇O₂max and velocity, which were systematically higher in the male subgroup. Since both predictors were positively associated with EC₀ in our data, their inclusion in the model led to lower predicted EC₀ values for male players and slightly higher values for females. This reflects physiological variation rather than an artifact of model inconsistency.

In general, running at higher velocities requires more energy per unit of time [49], but the energy cost per distance covered can be similar or even lower compared to running at slower speeds. Our data show that the energy cost of constant speed running increases at lower speeds, but the rate of increase decelerates beyond approximately 3.5 m/s, resulting in an almost plateau-like curve at higher velocities (Fig 4). This pattern appeared largely linear across the tested speed range, with no substantial deviation observed at lower velocities. Accordingly, we considered a linear model specification for EC_0_ to be appropriate and statistically justified. This is consistent with some previous studies investigating the relationship between running economy and speed [50–52], but also differs from one study [48] which showed a curvilinear U-shape with a nadir reflecting the most economical speed at 13 km/h (3.6 m/s). This study used highly trained endurance athletes specialized in long-distance running. Studies of EC_0_ in team sports have not investigated the influence of different velocities on EC_0_, they have all used a protocol consisting of constant speed running at one speed rather than constant speed running at different velocities [26–28].

### Practical relevance

The variation in the energy cost of running at constant speed across different sports and V̇O₂_max_ groups highlights the importance of using more than a single fixed value in the metabolic power approach. Instead, the selection of sport-specific EC₀ values is crucial, and an even more accurate approach would take into account individual differences in V̇O₂_max_. Given that athletes with a higher V̇O₂_max_ tend to have a higher EC₀, incorporating both sport type and individual physiological characteristics may improve the accuracy of metabolic power estimation. The metabolic power approach has been applied to several team sports, including soccer [3,9], handball [53,54], rugby league [10], Australian football [11] and field hockey [8]. However, sport-specific EC₀ values have only been established for soccer [17,26]. The inclusion of a sport-specific EC₀ is essential to refine and advance the metabolic power approach in each individual team sport, allowing for a more accurate assessment of energy expenditure. When individual EC₀ values are available, athlete monitoring can become even more precise and tailored to individual needs. Inaccuracies in the EC_0_ value can systematically affect the estimation of metabolic power and thus the cumulative metabolic work derived from tracking data. Even moderate deviations in the EC_0_ may result in substantial over- or underestimation of physical load, particularly in high-intensity intermittent sports [26]. This further underlines the value of using individualized or at least sport-specific EC_0_ values in applied athlete monitoring.

### Methodological considerations

Since each athlete completed only one of the three treadmill protocols, it was not possible to assess the isolated effect of protocol design (e.g., initial speed or stage duration) on the energy cost of running. As a result, potential protocol-related influences could not be disentangled from group characteristics such as sport type. In addition, our results need to be validated in a sport-specific context, taking into account factors such as running shoes and surface type, both of which may influence energy cost. Moreover, resting metabolic rate was not individually measured but assumed to be 3.5 ml/kg/min for all athletes. While this is a commonly accepted value in literature, individual variations in resting metabolic rate, especially in athletes with different body compositions and fitness levels, may introduce some degree of systematic bias. To evaluate the robustness of this assumption, we conducted a supplementary analysis using gross EC0. This model yielded nearly identical model fit and fixed-effect estimates, supporting the stability of our findings regardless of how resting V̇O_2_ is treated. Nonetheless, we chose to retain net EC_0_ in the main text to ensure comparability with previous studies, which have typically reported net energy cost values. Due to time constraints during routine performance diagnostics, a direct measurement of resting metabolic rate was not feasible. Furthermore, as data were collected over several years, minor variation due to equipment servicing or calibration cannot be ruled out. Additionally, one of the protocols included test stages starting at 2.0 m/s, which approaches the typical walk–run transition. All athletes were instructed to run throughout the test. These stages were retained in the model, and due to its non-linear structure, potential effects at lower speeds were appropriately accounted for.

### Perspective

This research is helping to improve the accuracy of the metabolic power approach, but there is still much work to be done. These values need to be compared with those obtained in sport-specific contexts and settings. Ideally, a single assessment per athlete at the beginning of each season would be sufficient to provide an individualized value. For a more comprehensive monitoring of energy expenditure during match play, it is essential to consider not only the energy cost of locomotion – calculated using the metabolic power approach – but also the additional demands of sport-specific actions. Future research should focus on the integration of these movement patterns to provide a more complete assessment of the total energy expenditure of an athlete’s in a competitive setting.

## Supporting information

S1 Table Model comparison results for different predictor combinations explaining the energy cost of constant speed running. Displayed are values for net and gross EC_0_, including deviance, log. Likelihood, AIC and BIC. Models were fitted as linear mixed-effects models with subject as random intercept. The final model (grey) model included velocity, velocity², V̇O_2_max and sport.
(DOCX)
