# Supplementary material for: Sport-specific variability in the energy cost of constant speed running: Implications for metabolic power estimations
Source: PLoS One. 2025 Aug 6;20(8):e0329323. doi: 10.1371/journal.pone.0329323 (PMC12327660; doi:10.1371/journal.pone.0329323)
Supplement: S1 Table — Displayed are values for net and gross EC0, including deviance, log. Likelihood, AIC and BIC. Models were fitted as linear mixed-effects models with subject as random intercept. The final model (grey) model included velocity, velocity², V̇O2max and sport. (DOCX) [file pone.0329323.s001.docx]

| Model | EC_0_-type | Fixed Effects | Random Effects | Deviance | log. Likelihood | AIC | BIC |
| --- | --- | --- | --- | --- | --- | --- | --- |
| Sports only | net | sport | Intercept (Subject ID) | 0.6 | -0.3 | 10.6 | 36.9 |
|  | gross | sport | Intercept | -102.8 | 51.4 | -92.4 | -66.5 |
| Sports + velocity | net | sport, velocity | Intercept + Slope (velocity) | -134.6 | 67.3 | -118.6 | -76.5 |
|  | gross | sport, velocity | Intercept + Slope (velocity) | -154.9 | 77.5 | -138.9 | -96.8 |
| Sports + velocity + velocity² | net | sport, velocity, velocity² | Intercept + Slope (velocity) | -147.4 | 73.7 | -129.4 | -81.9 |
|  | gross | sport, velocity, velocity² | Intercept + Slope (velocity) | -149.1 | 74.5 | -131.1 | -83.6 |
| sports + V'O_2_max | net | sport, V'O_2_max | Intercept | -211.8 | 105.9 | -195.8 | -154.5 |
|  | gross | sport, V'O_2_max | Intercept | -171.7 | 85.8 | -159.7 | -128.7 |
| Full model | net | sport, V'O_2_max, velocity | Intercept + Slope (velocity) | -226.7 | 113.4 | -208.7 | -162.2 |
|  | gross | sport, V'O_2_max, velocity | Intercept + Slope (velocity) | -259.9 | 129.9 | -241.9 | -195.4 |
| Full model + velocity² | net (Main text) | sport, V'O_2_max, velocity, velocity² | Intercept + Slope (velocity) | -266.9 | 133.5 | -246.9 | -195.3 |
|  | gross | sport, V'O_2_max, velocity, velocity² | Intercept + Slope (velocity) | -268.0 | 134.0 | -248.0 | -196.4 |
| Hockey players only + sex | net | sex, V'O_2_max, velocity | Intercept + Slope (velocity) | -184.5 | 92.2 | -168.5 | -131.7 |
|  | gross | sex, V'O_2_max, velocity | Intercept + Slope (velocity) | -191.3 | 95.7 | -175.3 | -138.6 |
| Hockey players only + sex + velocity² | net | sex, V'O_2_max, velocity, velocity² | Intercept + Slope (velocity) | -192.6 | 96.3 | -174.6 | -133.3 |
|  | gross | sex, V'O_2_max, velocity, velocity² | Intercept + Slope (velocity) | -192.2 | 96.1 | -174.2 | -132.8 |
| male only | net | sport, V'O_2_max, velocity | Intercept + Slope (velocity) | -98.0 | 49.0 | -80.0 | -39.8 |
|  | gross | sport, V'O_2_max, velocity | Intercept + Slope (velocity) | -124.2 | 62.1 | -106.2 | -65.9 |
| male only + velocity² | net | sport, V'O_2_max, velocity, velocity² | Intercept + Slope (velocity) | -125.1 | 62.6 | -105.1 | -60.4 |
|  | gross | sport, V'O_2_max, velocity, velocity² | Intercept + Slope (velocity) | -126.8 | 63.4 | -106.8 | -62.0 |

Table S1 Model comparison results for different predictor combinations explaining the energy cost of constant speed running. Displayed are values for net and gross EC_0_, including deviance, log. Likelihood, AIC and BIC. Models were fitted as linear mixed-effects models with subject as random intercept. The final model (grey) model included velocity, velocity², V̇O_2_max and sport
